# Evaluation of the brain activation induced by functional electrical stimulation and voluntary contraction using functional magnetic resonance imaging

**DOI:** 10.1186/1743-0003-9-48

**Published:** 2012-07-24

**Authors:** Kyung-Lim Joa, Yong-Hee Han, Chi-Woong Mun, Bong-Kyung Son, Chang-Hyung Lee, Yong-Beom Shin, Hyun-Yoon Ko, Yong-Il Shin

**Affiliations:** 1 Department of Rehabilitation Medicine, Pusan National University School of Medicine, Busan, South Korea; 2 Research Institute for Convergence of Biomedical Science and Technology, Pusan National University Yangsan Hospital, Busan, South Korea; 3 Department of Biomedical Engineering and, UHRC/FIRST, Inje University, Gimhae, South Korea; 4 Department of Radiology, Pusan National University School of Medicine, Busan, South Korea

## Abstract

**Background:**

To observe brain activation induced by functional electrical stimulation, voluntary contraction, and the combination of both using functional magnetic resonance imaging (fMRI).

**Methods:**

Nineteen healthy young men were enrolled in the study. We employed a typical block design that consisted of three sessions: voluntary contraction only, functional electrical stimulation (FES)-induced wrist extension, and finally simultaneous voluntary and FES-induced movement. MRI acquisition was performed on a 3.0 T MR system. To investigate activation in each session, one-sample *t*-tests were performed after correcting for false discovery rate (FDR; *p* < 0.05). To compare FES-induced movement and combined contraction, a two-sample *t*-test was performed using a contrast map (*p* < 0.01).

**Results:**

In the voluntary contraction alone condition, brain activation was observed in the contralateral primary motor cortex (MI), thalamus, bilateral supplementary motor area (SMA), primary sensory cortex (SI), secondary somatosensory motor cortex (SII), caudate, and cerebellum (mainly ipsilateral). During FES-induced wrist movement, brain activation was observed in the contralateral MI, SI, SMA, thalamus, ipsilateral SII, and cerebellum. During FES-induced movement combined with voluntary contraction, brain activation was found in the contralateral MI, anterior cingulate cortex (ACC), SMA, ipsilateral cerebellum, bilateral SII, and SI.

The activated brain regions (number of voxels) of the MI, SI, cerebellum, and SMA were largest during voluntary contraction alone and smallest during FES alone. SII-activated brain regions were largest during voluntary contraction combined with FES and smallest during FES contraction alone. The brain activation extent (maximum *t* score) of the MI, SI, and SII was largest during voluntary contraction alone and smallest during FES alone. The brain activation extent of the cerebellum and SMA during voluntary contraction alone was similar during FES combined with voluntary contraction; however, cerebellum and SMA activation during FES movement alone was smaller than that of voluntary contraction alone or voluntary contraction combined with FES. Between FES movement alone and combined contraction, activated regions and extent due to combined contraction was significantly higher than that of FES movement alone in the ipsilateral cerebellum and the contralateral MI and SI.

**Conclusions:**

Voluntary contraction combined with FES may be more effective for brain activation than FES-only movements for rehabilitation therapy. In addition, voluntary effort is the most important factor in the therapeutic process.

## Introduction

Upper extremity hemiparesis is the primary impairment underlying stroke-induced disability and is most frequently treated by therapists [1]. Only 20% of stroke survivors have normal upper extremity function 3 months later [2]. Periodic functional electrical stimulation (FES) promotes motor function recovery after a stroke [3,4], but the neural basis of this treatment is not fully understood at the level of the central nervous system. Besides the well-known peripheral effects on muscles themselves, the central therapeutic benefits of FES have been described in a few reports about central reorganization [5,6]. Previous studies also suggested that the FES effect is optimal when patterned electrical stimulation is delivered in close synchrony with attempted voluntary movement. Although functional neuroimaging studies have demonstrated that the clinical use of FES can activate the cerebral cortex [7], no reports have compared the effects of FES-induced cerebral cortex activation alone, voluntary contraction alone, and FES combined with voluntary contraction. Therefore, we investigated and compared these three treatments applied to wrist extensors in normal subjects using functional magnetic resonance imaging (fMRI).

## Subjects and methods

### Subjects

Nineteen healthy right-handed subjects who had no known neurological disorders (mean age ± standard deviation, 28 years ± 3.033) participated in the study after giving informed consent. The investigation complied with the Helsinki declaration and was approved by the local ethics committee.

### FES parameters and protocols

**Figure 1:**
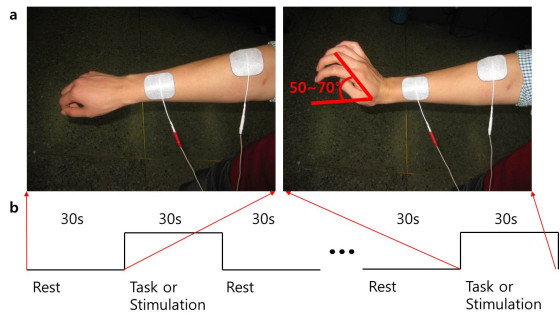
Experimental design.

FES was performed with a two-channel portable electrical stimulator (Cybermedic Corp., Iksan, Korea). We applied asymmetric, biphasic, charge-balanced, rectangular pulse shapes. The rising and falling times were 0.5 s, and the stimulation and rest times were 1 s. The depolarizing pulses had a width of 200 μs and a frequency of 20 Hz. The wrist extensor muscles (i.e., extensor carpi radialis longus, extensor carpi radialis brevis, extensor digitorum communi, and extensor carpi ulnaris) were stimulated by a stimulating channel with a Synapse self-adhesive electrode (30 mm X 30 mm; Ambu A/S, Baltorpbakken 13, 2750 Ballerup, Denmark). The stimulation intensity was adjusted to produce wrist extension in the range of motion between 50–70 degrees within the limit in which the subject felt no discomfort (Figure 1). Some subjects felt discomfort and needed lower amplitudes. The resulting stimulation current amplitudes were in the range of 16–20 mA.

### fMRI experimental protocols

The fMRI protocol had three stimulation sessions: voluntary contraction alone, FES alone, and FES combined with voluntary contraction. During each task or stimulation session, experimental blocks consisting of control (resting stage) and task or stimulation period were repeated four times with 10 scans each time. The experimental blocks consisted of four rest periods and four stimulation periods (30 s each). Voluntary contraction was performed at the same speed and manner as the FES-only protocol. FES combined with voluntary contraction was performed to make maximal voluntary wrist contraction while FES was applied at the same speed and manner as the FES-only protocol.

Each subject was asked to lie down in the supine position and to look at a computer monitor displaying instructions. The head was secured and movement was minimized with foam padding. The arms were supported if subjects were uncomfortable, and the right elbow was fixed, which only allowed hand movement. FES stimulation was triggered with a push-button switch that was controlled outside of the MRI room.

### fMRI acquisition and analysis

Blood oxygenation level-dependent (BOLD) fMRI, which detects neural activity, was performed in a 3.0 T magnetic resonance imaging scanner (Verio, Siemens, Erlangen, Germany) with a 12-channel head coil. T1-weighted images were obtained using a magnetization-prepared rapid acquisition gradient recalled echo (MPRAGE) pulse sequence for gross anatomic visualization and co-registration. BOLD images were obtained using the following echo planar imaging (EPI) sequence: repetition time (TR) = 3000 ms, echo time (TE) = 21 ms, flip angle = 90°, field of view (FOV) = 19.2 cm, matrix size = 64 mm × 64 mm and slide thickness = 3 mm.

Functional image analysis was performed using the SPM8 (http://www.fil.ion.ucl.ac.uk/spm) software package on the MATLAB (The Mathworks, Natick, MA, USA) platform. Images from each session were realigned using affine transformation to correct for head motion. Realigned images were co-registered with T1 anatomical images, and then normalization was conducted using the 152 Montreal Neurological Institute (MNI) template. To increase the signal-to-noise ratio (SNR), smoothing was performed using a Gaussian kernel with an 8-mm full-width half maximum (FWHM). Preprocessed images from each session were regressed by the time series hemodynamic response function (HRF) and then correlated. For comparison within each session, one-sample *t*-tests were performed after correcting for the false discovery rate (FDR; < 0.05) to compensate for type 1 errors, in addition to identifying statistical differences between FES-induced alone and combined movement. Two-sample *t*-tests were performed using a contrast map, which was analyzed by one-sample *t*-tests.

## Results

**Figure 2:**
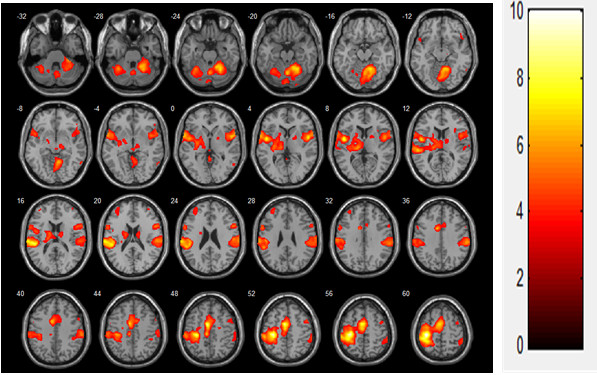
Voluntary contraction (N = 19, t(18) > 3.141, FDR < 0.05).

**Figure 3:**
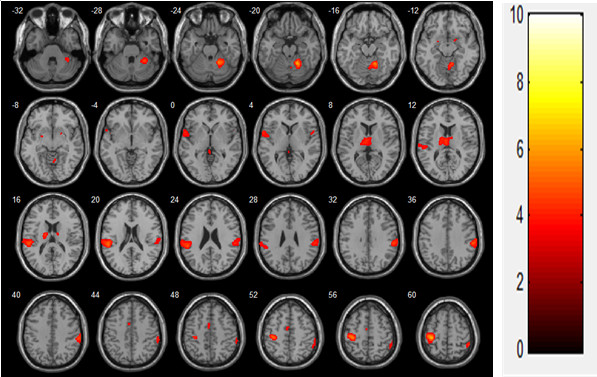
FES-induced stimulation (N = 19, t(18) > 3.116, FDR < 0.05).

**Figure 4:**
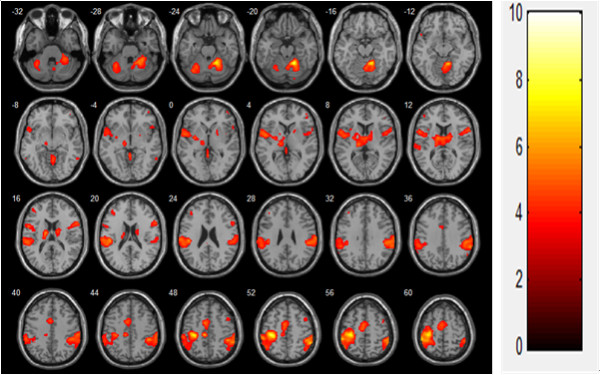
FES combined voluntary contraction (N = 19, t(18) > 3.961, FDR < 0.05).

In the voluntary contraction alone condition, brain activation was observed in the contralateral primary motor cortex (MI), thalamus, bilateral supplementary motor area (SMA), primary sensory cortex (SI), secondary somatosensory motor cortex (SII), caudate, and cerebellum (mainly ipsilateral; Figure 2, Table 1). During FES-induced wrist movement, brain activation was observed in the contralateral MI, SI, SMA, thalamus, ipsilateral SII, and cerebellum (Figure 3, Table 2). During FES-induced movement combined with voluntary contraction, brain activation was found in the contralateral MI, anterior cingulate cortex, SMA, ipsilateral cerebellum, bilateral SII, and SI (Figure 4, Table 3).

**Table 1:** Activation region in voluntary contraction only movement

**Cluster**	**functional region**	**Contralateral**					**Ipsilateral**				
		**x**	**y**	**z**	**Max t**	**No.Voxels**	**x**	**y**	**z**	**Max t**	**No.Voxels**
**PrCG**	**MI**	−34	−26	62	12.97	1087					
**SFG**	**SMA**	−16	−5	71	7.91	986	6	4	66	3.43	356
**PoCG**	**SI**	−36	−45	63	7.40	1556	62	−26	36	5.77	684
	**SI**						62	−26	20	4.89	416
**RO, STG**	**SII**	−46	0	6	7.02	285	50	2	6	5.48	202
	**SII**	−60	8	0	6.37	257	58	8	2	5.36	106
**IFG**		−58	6	28	4.72	125	58	12	22	4.81	148
**Thalamus**		−14	−20	8	5.00	184					
**Caudate**		−12	−6	16	4.12	25	14	−8	18	3.65	
**Pallidum**		−26	−12	0	3.91	5					
**Cerebellum**		−34	−54	−26	5.39	208	20	−54	−46	6.74	2656

Coordinates depict voxel with the highest t-value.

Max t: maximum t-score; No. Voxels:, number of voxels; PrCG: primary central gyrus;

SFG: superior frontal gyrus; PoCG: post central gyrus;RO:rolantic operculum;

STG: superior temporal gyrus; IFG: inferior frontal gyrus; MI: primary motor cortex;

SMA: supplementary motor area; SI: primary somatosensory cortex;

SII: secondary somatosensory cortex.

**Table 2:** Activation region in FES only movement

**Cluster**	**functional region**	**Contralateral**					**Ipsilateral**				
		**x**	**y**	**z**	**Max t**	**No.Voxels**	**x**	**y**	**z**	**Max t**	**No.Voxels**
**PrCG**	**M1**	−36	−28	62	6.62	300					
	**SMA**	−20	−16	76	3.54	41					
**Cerebellum**							22	−52	−20	5.89	370
							22	−52	−50	4.30	49
							16	−58	−50	4.26	73
**PoCG**	**S1**	−54	−28	20	5.02	237					
**SFG**	**SMA**	−12	−10	78	4.28	34					
**SMG**	**SII**						60	−28	34	4.27	178
**Thalamus**		−14	−8	14	4.22	103					
		−4	−12	10	4.21	4					

Coordinates depict voxel with the highest t-value.

Max t: maximum t-score; No. Voxels: number of voxels; PrCG: primary central gyrus;

PoCG: post central gyrus; SFG: superior frontal gyrus; SMG:supramarginalgyrus;

MI: primary motor cortex; SMA: supplementary motor area;

SI: primary somatosensory cortex; SII: secondary somatosensory cortex.

**Table 3:** Activation region in FES combined voluntary contraction

**Cluster**	**functional region**	**Contralateral**					**Ipsilateral**				
		**x**	**y**	**z**	**Max t**	**No. of Voxels**	**x**	**y**	**z**	**Max t**	**No. of Voxels**
**PrCG**	**MI**	−34	−26	52	8.47	786					
	**SMA**	−34	−28	64	8.32	691					
**PoCG, IPL**	**SI**	−50	−36	58	6.11	1107	56	−46	52	5.07	292
	**SI**	−62	−22	24	4.93	986					
**Cerebellum**		−34	−68	−24	5.16	253	18	−50	−18	7.50	1219
							30	−44	−28	5.24	136
							4	−62	−16	4.74	54
**RO, SMG, PoCG**	**SII**	−54	−28	18	4.81	194	54	−30	34	5.36	871
	**SII**	−48	0	6	4.77	434	50	4	8	3.78	943
	**SII**	−54	8	14	4.36	244	64	−40	36	4.99	292
**Insula**		−46	−24	22	5.95	842					
**ACC**		−8	−26	48	5.73	73					
**Thalamus**		−12	−8	14	4.84	315					
		−14	−20	8	4.76	257					
		−4	−8	10	4.13	16					
**IFG**							60	14	18	4.42	184

Coordinates depict voxel with the highest t-value.

Max t: maximum t-score; No. Voxels: number of voxels; PrCG:precentralgyrus;

PoGG:postcentralgyrus; ACC: anterior cingulate cortex; IPL: intra-parietal lobule;

RO:rolanticopeculum; SMG:supramarginalgyrus; IFG: inferior frontal gyrus;

MI: primary motor cortex; SI: primary somatosensory cortex;

SII: secondary somatosensory cortex.

**Figure 5:**
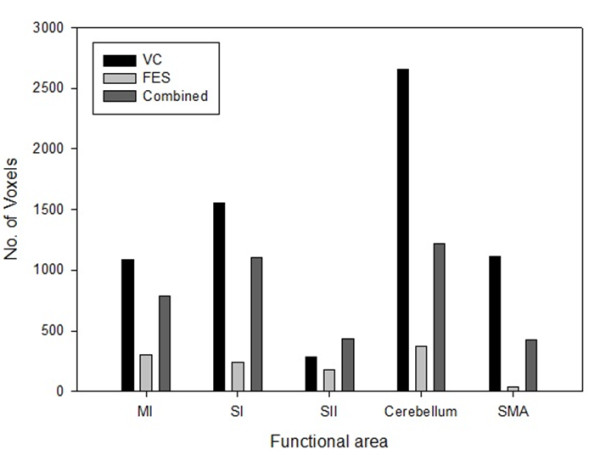
** Comparison of brain activation areas during three stimulation sessions.** Abbreviations: No. Voxels, number of voxels, VC, voluntary contraction, FES, functional electrical stimulation, MI, primary motor cortex, SI, primary somatosensory cortex, SII, secondary somatosensory cortex, SMA, supplementary motor area.

**Figure 6:**
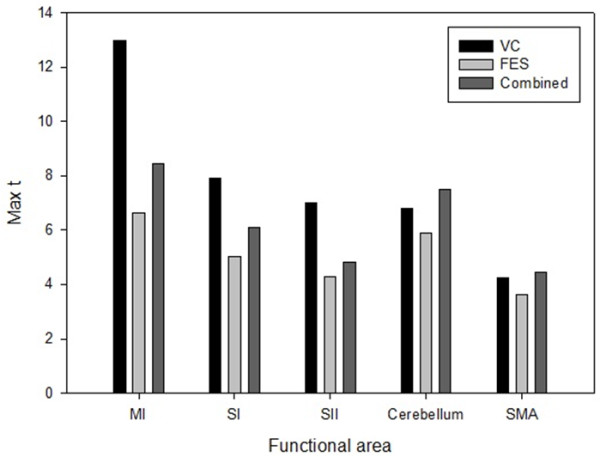
** Comparison of brain activation extents during three stimulation sessions.** Abbreviations: Max t, maximum t-score, VC, voluntary contraction, FES, functional electrical stimulation, MI, primary motor cortex, SI, primary somatosensory cortex, SII, secondary somatosensory cortex, SMA, supplementary motor area.

**Figure 7:**
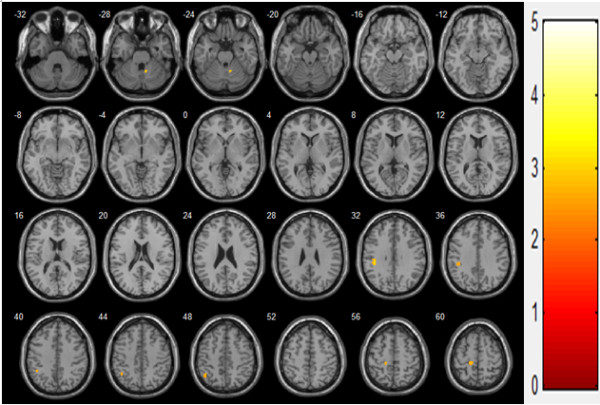
** Combined movement > FES only movement (N = 38, t(36) > 3.333,*p*** **< 0.001).**

The activated brain regions (number of voxels) of the MI, SI, cerebellum, and SMA were largest during voluntary contraction alone and smallest during FES alone. The activated brain regions of the SII were largest during voluntary contraction combined with FES and smallest during FES contraction alone. The brain activation extent (maximum *t* score) of the MI, SI, and SII was largest during voluntary contraction alone and smallest during FES alone. The brain activation extent of the cerebellum and SMA during voluntary contraction alone was similar during FES combined with voluntary contraction; cerebellum and SMA activation during FES movement alone was smaller than voluntary contraction alone or voluntary contraction combined with FES (Figures 5 and 6). Between FES movement alone and combined contraction, activated regions and extent of combined contraction was significantly higher than that of FES movement alone in the ipsilateral cerebellum, contralateral MI, and SI (Figure 7, Table 4).

**Table 4:** More activation region in combined movement than FES only movement

**Cluster**	**functional region**	**Contralateral**					**Ipsilateral**				
		**x**	**y**	**z**	**Max t**	**No.Voxels**	**x**	**y**	**z**	**Max t**	**No.Voxels**
**PrCG**	**M1**	−14	−30	58	2.85	58					
**Cerebellum**							10	−54	−26	3.00	30
**PoCG**	**S1**	−40	−32	32	3.05	77					
**IPL**	**S1**	−42	−56	46	2.67	54					

Coordinates depict voxel with the highest t-value.

Max t: maximum t-score; No. Voxels: number of voxels; PrCG: primary central gyrus;

PoCG: post central gyrus; MI: primary motor cortex; SI: primary somatosensory cortex; IPL: intra-parietal lobule.

## Discussion

We compared the effects of three different fMRI paradigms (i.e., FES alone, voluntary contraction alone, and FES and voluntary contraction combined) on cortical activation, and our results demonstrated that five distinct brain regions (i.e., MI, SI, SMA, SII, and cerebellum) contributed to active and passive motor tasks [8] and showed significant and reproducible activation. Strong activation was elicited in the MI and SI and extended to the same degree in both somatosensory and motor areas adjacent to the central sulcus. These areas were closely connected to each other, and sent efferent inputs to and received afferent outputs from the distal extremities. Their co-activation was expected due to the nature of our stimulation and the motor output it produced.

Primary motor cortex activation have been observed with passive movement, even without electrical stimulation [9], and recent functional neuroimaging studies have demonstrated that electrical stimulation or proprioceptive inputs can activate the M1 and S1 [10]. The M1 receives afferent inputs via the SI or thalamus [11,12]. Increased motor cortical excitability may then facilitate greater voluntary activation of the relevant neuronal network, thereby leading to improved function [13].

In the present study, there was additional activation of the contralateral SMA in subjects in all three stimulation sessions (voluntary contraction alone, FES contraction alone, and FES combined with voluntary contraction). Although the SMA is associated with motor planning of the performance of a complex motor task [14], it also receives somatosensory inputs and is activated by passive movement [15,16]. As seen in previous studies, the SMA was activated by FES through passive movement and somatosensory inputs. Significant increases in activity were found in the SII bilaterally during all three stimulation sessions. SII activity has also been reported in other electrical stimulation paradigms. For example, Backes et al. [17] stimulated the median nerve while participants performed a selective attention task and observed enhanced secondary somatosensory activity that they ascribed to selective attention. Secondary somatosensory areas are considered sites for higher-order processing of sensory information and sensorimotor integration [18]. Bilateral activity in such areas is observed when brushing the fingers and the palm of the hand with a rough sponge [19], when vibration stimulation is applied to the left thumb [20], during median nerve stimulation [17], and wrist flexion/extension stimulation [21]. This may explain the larger activated brain regions during voluntary contraction combined with FES rather than during voluntary contraction alone.

We also found that cerebellum activation was mainly ipsilateral. This pattern of cerebellar activation is in line with those of other studies using passive movements [9]. A previous study using lower extremity electrical stimulation indicated that afferent spinocerebellar information from muscles, joints, and tactile receptors is important for the preparation and correction of ongoing movement [22]. Takanashi et al. [23] also demonstrated a similar somatotopical organization within the cerebellum after electrical stimulation. It is conceivable that the cerebellar activation pattern observed in the present study was elicited by passive movement, as well as by the electrical stimulation itself.

The activated brain regions of the MI, SI, cerebellum, and SMA were largest during voluntary contraction alone and smallest during FES alone. The brain activation extent of the MI, SI, and SII was largest during voluntary contraction alone and smallest during FES alone. These results suggest that FES combined with voluntary contraction can enhance the cortical plasticity resulting from the cumulative effects of FES-induced cortical excitability together with excitability due to voluntary contraction. FES produces changes in cortical excitability [22]. Furthermore, the repetition of even a simple movement can produce changes in cortical excitability that leads to transient reorganization in motor connectivity [24].

The smallest brain activation areas and extent during FES contraction alone may be explained in other ways. In a study regarding the reproducibility of cerebral sensorimotor activation with fMRI, Loubinoux et al. [25] observed significant changes in signal intensity and decreases in brain activation between the first and second fMRI sessions. These changes were marked in the contralateral MI, SI, SMA, cigulum, and parietal cortex. They also conducted an experiment on the brain activation test–retest effect during active and passive movement [25] and reported that changes in brain activation areas and extent were marked during passive movement but not during active movement. In the present study, FES contraction alone was performed after voluntary contraction alone. Passive wrist movement with FES may be the reason why brain activation areas and extent were smallest with repetitive fMRI.

As previously mentioned, stimulation was well tolerated, although not comfortable for all subjects. Despite the non-painful nature of the stimulus, activation within the cingulate was observed during FES combined with voluntary contraction. This may be explained in that some subjects felt discomfort but did not require reduced amplitude, while others did. This also means that current intensity was set differently for each subject to avoid painful sensations. We focused on maximum wrist extension and thus we used different current intensities for each subject to induce maximum movement without pain. This FES application method is currently used for rehabilitation therapy in rehabilitation centers; we applied FES to mimic the current method of FES therapy for stroke patients. Previous fMRI studies on FES-induced wrist movement did not standardize FES during wrist movement. Thus, standardization of electrical stimulation was not essential in this study.

In rehabilitation clinics, FES combined with voluntary contraction is used as a new hybrid FES therapy in stroke patients [19]. Grisenko et al. [26] reported that FES-assisted exercise therapy improved hand function in patients with hemiplegia whose level of motor function excluded them from constraint-induced movement therapy. They also found that cerebral blood flow in the sensory-motor cortex area on the injured side increased during the power-assisted FES session compared to simple active movement or simple electrical stimulation in a multichannel near-infrared spectroscopy study to noninvasively and dynamically measure hemoglobin levels in the brain during functional activity. This suggests sensory components as a possible mechanism for motor improvement in FES therapy.

However, the present study has several limitations. First, the subject group was unbalanced, as it consisted of 19 males and 4 females. Because the number of female participants was small and the results for the female group were significantly different from those for the male group, females were not included in the statistical analyses. Therefore, we analyzed the fMRI data using only the male group throughout. Second, a force measurement during voluntary contraction and voluntary contraction combined with FES was not carried out. If we measured force during maximum voluntary contraction and had subjects do voluntary contraction with the same power during FES combined with voluntary contraction, the results may have been more persuasive. Finally, because the three stimulation sessions were not randomized, we do not know whether session order influenced the results.

The most important factor in brain rehabilitation is not the activated pathway per se, but rather cortical activation itself. Brain plasticity refers to the brain’s ability to change structures and functions altered by the environment. Therefore, the principal rehabilitation procedure for brain-injured patients is environmental manipulation [27]. By combining patterned electrical stimulation with attempted voluntary movement, the movement may be interpreted more as the patient’s own and, like ordinary voluntary movement, may co-activate more motor control regions and sensory areas. This may be crucial in motor recovery at the brain level. The present study showed that the clinical use of FES has a direct effect on the cerebral cortex, and that FES in combination with voluntary training may enhance the benefits of the standard FES therapy.

## Conclusion

Neuromuscular stimulation combined with voluntary movement produces more brain activation areas and extent than neuromuscular stimulation alone. These results suggest that, for rehabilitation therapy, voluntary contraction combined with FES may be more effective for brain activation than FES movement alone and may provide the basis for FES-related research for understanding mechanisms of neural plasticity or reorganization in motor function recovery.

## Competing interests

The authors declare that they have no competing interests.

## Authors’ contribution

YH and BK carried out the fMRI studies, KL, CY, and YI participated in the study design and drafted the manuscript. All authors read and approved the final manuscript.
